# Group therapy for problematic chemsex in Ngos community treatment settings in Spain

**DOI:** 10.1192/j.eurpsy.2024.853

**Published:** 2024-08-27

**Authors:** J. Curto Ramos, N. Sanz Velasco, L. Carballeira, I. López Álvarez, P. Barrio, L. Ibarguchi, A. García, H. Dolengevich Segal

**Affiliations:** ^1^Department of Psychiatry, Clinical Psychology and Mental Health, La Paz University Hospital; ^2^ Apoyo Positivo; ^3^Department of Psychiatry, Hospital Clínico San Carlos; ^4^Department of Psychiatry, Hospital Doce de Octubre, Madrid; ^5^Department of Psychiatry, Hospital Universitario Marqués de Valdecilla, Santander; ^6^Dual Disorders Program. Department of Psychiatry, Henares University Hospital, Madrid, Spain

## Abstract

**Introduction:**

The intentional use of drugs before or during sexual intercourse (chemsex) is a phenomenon of special importance in the MSM (men who have sex with men) population due to its impact on mental, physical and sexual health. Group therapy has been included in several programs for chemsex users.

**Objectives:**

To describe and to compare the different group therapy treatments for problematic chemsex users in NGOs community treatment settings in Spain.

**Methods:**

We conducted several interviews with key informants from 5 NGO in Spain. A qualitative analysis of the different group therapy treatments for problematic chemsex was performed.

**Results:**

Different models of groups were described including: psychoeducational, support, interpersonal process, harm reduction and mindfulness-based cognitive groups. Most of the group interventions developed were support and psychoeducational based. There were fewer interpersonal group and relapse prevention group therapy. The different models of group intervention were considered useful and necessary for deliver information in a culturally sensitive context and for reducing drug use, social isolation and loneliness.

**Conclusions:**

Chemsex is a phenomenon that needs a multidisciplinary approach, including individual and group therapy. Group therapy for problematic chemsex has several advantages over individual model treatments, including the reduction of sense of isolation, loneliness, information and feedback from peers. More research is needed to analyze the implementation and efficacy of group therapy for chemsex users in different contexts.

**Disclosure of Interest:**

None Declared

